# Charitable giving and reflexive individuals: How personal reflexivity mediates between structure and agency

**DOI:** 10.1177/0539018416646486

**Published:** 2016-07-08

**Authors:** Balihar Sanghera

**Affiliations:** School of Social Policy, Sociology and Social Research, University of Kent, Canterbury, UK

**Keywords:** agency, charitable giving, reflexivity, sentiments and discourses, agentivité, pratique de la charité, réflexivité, sentiments et discours

## Abstract

This article examines how individuals are reflexive beings who interpret the world in relation to things that matter to them, and how charitable acts are evaluated and embedded in their lives with different degrees of meaning and importance. Rather than framing the discussion of charitable practices in terms of an altruism/egoism binary or imputing motivations and values to social structures, the article explains how reflexivity is an important and neglected dimension of social practices, and how it interacts with sympathy, sentiments and discourses to shape giving. The study also shows that there are different modes of reflexivity, which have varied effects on charity and volunteering.

## Résumé

L’article examine en quoi les individus sont des êtres humains réflexifs qui interprètent le monde en relation aux choses qu’ils jugent importantes, et comment les pratiques de la charité sont évaluées et imbriquées dans leurs vies à des degrés divers de signification et d’importance. Plutôt que de cadrer la discussion des pratiques charitables en termes d’opposition binaire altruisme/égoïsme ou encore d’imputer les motivations et les valeurs aux structures sociales, l’auteur examine en quoi la réflexivité est une dimension importante et négligée des pratiques sociales, et comment elle interagit avec la sympathie, les sentiments et les discours pour donner forme à l’acte charitable. L’étude montre aussi qu’il y a différents modes de réflexivité, qui ont des effets divers sur la pratique de la charité et le volontariat.

How does personal reflexivity mediate between structure and agency in shaping charitable giving? Individuals are reflexive beings, who interpret the world in relation to things that matter to them, deliberating and prioritizing elements of their life that are of key concern, such as physical well-being, practical worldly achievements, family happiness, emotional relationships, social self-esteem, political and moral values, and faith (Archer, 2012: 102–111; Taylor 1989: 62–63). The article will argue that people make evaluative decisions about charitable giving based on their concerns and life situations (Stirling, 2010). It will also discuss how gendered roles, moral individualism and class subjectivities can shape practices of giving.

Archer (2003: 135–150) argues that first-person reflexivity is indispensable, enabling individuals to navigate their way through the world. People have internal conversations to discern things of importance, to dedicate themselves to their goals and to design courses of action. Internal conversations consist of various mental activities, including prioritizing goals, planning the day or week, having imaginary conversations, rehearsing speeches and performances, re-living past periods and events, imagining the future, mulling over problems, and clarifying issues and situations. People assess what social factors constrain and enable their life projects in a world not of their making, how much endurance is needed to stay the course and what to do next (Archer, 2000: 230–241). Reflexivity is personal and subjective, and has causal powers. It is also about real things, deliberating (always fallibly) about social relations and objective powers that affect individuals’ goals (Archer, 2007: 15–16).

Porpora (1989) argues that much of contemporary sociology tends to conflate the distinct properties and powers of structure and agency to produce reductionist theoretical frameworks. For instance, in Bourdieu’s (1990) account of social practices, the key concept of habitus conflates structure and agency, as it is both structured and structuring, a product of social structural position, and it shapes thoughts and actions. Habitus consists of durable dispositions (i.e. ways of acting, seeing and making sense of the world) that operate below the level of consciousness. Actors operate according to an implicit practical logic and a ‘feel for the game’ that enable them to deal with a wide variety of situations in predictable ways without consciously thinking about rules. Yet Bourdieu also maintains that actors strategize in the social field, which surely requires reflexivity, though Bourdieu tends to evade this implication. There is also a tendency in Bourdieu’s work to assume an ontological complicity between habitus and field, implying a high degree of adaptability on the part of the subject. But as Sayer (2005: 35) notes, this means that actors become skilled at playing games rather than evaluating them, and they want the world and its games to be different. Internal conversations make sense of people’s relationship to the world, which is not simply one of accommodation but also of resistance. They are able to imagine and desire a different world.

Furthermore Sayer (2005: 42) argues that, while Bourdieu provides an insightful understanding of how people judge themselves and others, and the practices and objects associated with them, their evaluations are strategic, functional and aesthetic rather than ethical. Bourdieu’s treatment of disinterested moral judgements has been criticized as inadequate and problematic (Lamont, 1992). Despite warning his readers not to misread him as offering a reductionist, economistic and cynical narrative of symbolic exchanges, ‘hermeneutics of suspicion’ loom large in his work (Bourdieu, 2000: 191–202). Caillé (2001) argues that, although Bourdieu becomes preoccupied with the issue of disinterestedness as a possibility, there is little real change in his theoretical vision, because disinterestedness is still conceived as merely illusory. Moreover, as Sayer (2010) maintains, moral judgements are distinct from aesthetic and practical ones, because morality has universalizing qualities that cut across class boundaries.

Philanthropic studies also tend to conflate structure and agency, producing reductionist accounts of charitable giving where social and cultural structures determine actors’ thinking and practices (see Bekkers & Wiepking, 2011 for a review of the literature). Pro-social behaviours motivated by empathy, altruism and generosity are sometimes imputed to class structures. For instance Collins and Hickman (1991) argue that the major reward the upper classes receive in participating in the voluntary sector is the cultural status of being a known altruistic person. Charitable giving allows the upper classes to legitimize their high social status. Ostrower (1998) identifies how rich donors’ attitudes and practices at philanthropic events reinforce class boundaries, so that elite philanthropy produces class cohesion. Breeze (2013) suggests that giving is not shaped by recipients’ needs but by donors’ tastes and preferences, which are acquired as a result of socialization and lifelong experiences, such as family and social upbringing, education and social networks. Working-class donors may refuse to support charities that promote social causes and cultural activities (such as opera and ballet) that are incongruent with their identity, and that they perceive to be relevant only to other classes. But as a result of embedding charitable giving in class structures, these studies tend to deny actors the powers to think and act independently of their class positions.

The present article has five sections. The first section will examine the different ways in which reflexivity can mediate structure and agency. In the second section, I will describe the research design and methods. The third section will discuss the findings on how the different modes of reflexivity shape giving. I will address some possible criticisms of the study in the fourth section. Finally, I will make some concluding remarks.

## The three-stage model of reflexive donors and volunteers

In explaining social practices, critical realists (such as Fairclough & Fairclough, 2010; Porpora, 1989; Sayer, 2005) acknowledge both the effects of pre-existing social structures (e.g. class-based life chances, gender roles, social norms, organizations and discourses) and the ability of actors to intentionally bring about change in light of their personal concerns and goals. Archer (2007: 5–22) develops this view to argue that the two separate entities of structure and agency are mediated by personal reflexivity, so that courses of action are produced through reflexive deliberations of agents, who subjectively (and always fallibly) determine their life goals and projects in relation to their objective circumstances. The standard two-stage model of structure and agency is rejected in favour of the three-stage model, in which internal conversations mediate the impact of social structures on agents’ concerns and condition individual responses to particular social situations. Without reflexivity there would be no explanation of what people actually do and how they modify their projects in terms of contextual feasibility. Reflexivity enables people to accept, evade or resist social expectations, to negotiate clashing expectations and to deal with contingencies.

Archer (2012: 12–41) further argues that, even though reflexivity is a regular exercise of the mental ability shared by all people (with some exceptions), it is exercised in different ways depending on the relations people establish with their social contexts and their dominant concerns. Her empirical studies (2003, 2007, 2012) identify four modes of reflexivity. First, *communicative* reflexivity refers to internal conversations that need to be completed and confirmed by others before they lead to courses of action. Second, *autonomous* reflexivity is characterized by self-contained inner conversations that lead directly to action. Third, *meta*-reflexivity is exercised by individuals who are critically reflexive about their own internal conversations and the prospects of effective action in society. Whereas autonomous reflexives are primarily reflexive about the means to their ends, where their ends are not problematized, meta-reflexives worry about what their ends should be. Fourth, *fractured* reflexivity is exercised by individuals who cannot conduct purposeful internal conversations and thus cannot design effective courses of action.

While people engage in one or more modes of reflexivity over the course of their lives, Archer (2007: 90–99) argues that they tend to establish a dominant mode of reflexivity that collectively contributes to the structuring of society. Individuals whose internal dialogues are predominantly characterized by communicative reflexivity have key features of contextual continuity and social stability that cement social relationships and local networks. As communicative reflexives require others’ input to complete their deliberations, they tend to have strong familial and social ties and to stay put in the locality, collectively contributing to social stability and value consensus. It is their relationships that matter most to them, whether at home, university, work or elsewhere (Sayer, 2009). While people who largely have autonomous reflexive internal conversations also exercise communicative reflexivity and accommodate the interests of others, they are oriented towards practical worldly outcomes, self-development and enterprise. Their practical activities involve extensive solitary practice and reflexive monitoring. Their key features are contextual discontinuity and social mobility, which together produce social dynamism and productivity. In contrast to autonomous reflexives’ self-confidence, individuals who predominantly exercise meta-reflexivity are self-critical and tend to be preoccupied with the moral worth of their projects and their worthiness to undertake them (Scambler, 2012). Meta-reflexives’ key features are contextual incongruity and social change, which collectively contribute to social criticism and activism. Not surprisingly, as moral critics, they are likely to be more involved in civil society than others. People whose internal dialogues are dominated by fractured reflexivity lack the stability, self-esteem and vision of other reflexives, and are unable to purposefully plan their life projects. They are inclined to be socially passive and disoriented as a result of unsuccessful reflexive deliberations.

Since the 1980s, charity and voluntary action have become more widespread, partly reflecting a neoliberal strategy to reduce state responsibility for the provision of public goods. There are over 191,000 registered charities in the UK (Mohan & Breeze, 2016: 6). But who participates, and why do they donate to or join one organization rather than another? Archer (2007: 311–313) believes that only meta-reflexives are committed to the voluntary sector, and that other groups are unresponsive and passive. She (2007: 282, 298) argues that communicative reflexives tend to be uninterested in joining in social activities outside their circle of friends, and are indifferent towards civil-society organizations, and that autonomous reflexives are too preoccupied with their own solitary practices to participate in charities.

While this article will use Archer’s ideas on reflexivity, it will challenge her claims about who participates in civil society. I will maintain that communicative reflexives can regard charities as an opportunity to do good work with and for significant others and to affirm communal values. Their sympathy and compassion beyond their micro life-worlds of intense relationships are likely to be restricted to familiar groups and causes. Autonomous reflexives can obtain personal pleasure from their charitable performances and achievements. Charities and associations also have an instrumental value, helping them to pursue practical worldly outcomes. Meta-reflexives are often sensitive to issues of fairness, justice and suffering. They breathe life into political parties and faith-based organizations, and participate in civil society, in the hope of bringing about change. Fractured reflexives tend not to engage in voluntary giving and action, because they are unable to successfully deliberate on charitable giving.

## Research design and methods

Between 2008 and 2009, I conducted in-depth interviews with 41 individuals from a range of occupations, including public-sector administrators, university lecturers, social care workers, home-keepers, self-employed workers, mature students and retirees. Most of the interviews were conducted in Kent, UK. Many interviewees were recruited through mass emails to several local public and charity organizations, asking for volunteers to participate in a research project on giving and volunteering. All those who responded were subsequently interviewed. Several interviewees known to me were approached in order to have more ‘black’ and middle-class donors in the sample. As one of the aims of the research was to delineate a new mechanism of reflexivity that operates in social life, the sample was skewed towards donors and volunteers who possibly have reflected on giving more than others, and had more to say about it.

The semi-structured interviews consisted of two parts, lasting on average two and a quarter hours. The first part asked the interviewees to recount their life history, describing their life from their early upbringing and schooling through to their current family and work situation, their personal goals and routines. Interview questions included: Can you please tell me something about yourself from childhood and schooling to adult life? What were the important events or who were key people in your life? Can you please describe your typical day and week? Typically over a month or a year, can you say what do you do together with your neighbours / people on your street / members of the local community? Or maybe what you do for them? This part of the interview aimed to grasp how the interviewees understood and interpreted their own life; more specifically, what were their key concerns, what were they attentive to, how did these things change over time, and how were charitable acts embedded into their daily, weekly or monthly routines.

In the second part, interviewees were asked to recall their acts of giving and volunteering, and to describe their feelings and motivations. Interview questions included: Recall an incident when you gave money or time to a charity, a cause or someone to help out, talk me through how it began, what were you thinking and feeling. What were the reasons and motivations for this particular action and its timing? Can you say something about whether your friends, family members and work colleagues give money or volunteer? Of the money and time you have given to things, causes or people, which one has meant the most and the least to you? Can you say why giving or helping out matters to you? This part of the interview aimed to grasp how charitable acts are understood and interpreted, what reasons and motivations were given for them, and how other people shaped their donations. Overall, a picture emerged of how interviewees navigated their way through the world, being attentive to things of importance, to their well-being, and to what extent charitable giving was a meaningful and significant activity in their lives.

The interviewees were assigned a social class using multi-dimensional criteria: social-class upbringing (working- or middle-class parents), educational qualifications (school, college or university), occupation (unskilled, semi-skilled, skilled or professional) and household economic situation (struggling to make ends meet, managing to cope or having a comfortable lifestyle). In addition some interviewees defined themselves into a particular social class. Based on the interview data, the sample consisted of 19 working-class people, 16 lower-middle-class and six upper-middle-class.

In terms of gender composition, 25 women, 15 men and one transgender person participated in the research. The sample also consisted of five ‘black’ interviewees (three British Asians, one African immigrant and one Iranian immigrant) and three ‘white’ immigrants (one Argentinean, one Greek Cypriot and one South African). The rest were white British. The study also had seven retirees, of whom one was a part-time lay clergy and three were involved in managing local civic associations (a table-tennis club, a residents’ housing association and a naval heritage charity). There were two young undergraduates just about to complete their degrees. Most interviewees were young or middle-aged adults, and a few were approaching retirement.

All interviews were digitally recorded, and interviewees were reassured about confidentiality and anonymity. The interviews were transcribed, and then the transcripts were returned to them for checking and editing. Only a few made slight alterations to the text, correcting minor factual details. The subsequent analysis was coded using various labels, which emerged after reading the transcripts a couple of times. Some labels, such as ‘giving money’, ‘giving time’, ‘values’, ‘faith’, ‘tithes’, ‘caring for others’, ‘character’, ‘reflections upon giving’, ‘informal giving’ and ‘why giving matters’, identified key themes shared across the transcripts. Other labels, including ‘justice’, ‘activism’, ‘strategic giving’, ‘self-interest’, ‘family and children’ and ‘sympathy’, were more evident in some transcripts than others. Categorizing interviewees into different modes of reflexivity was done in a looser way than Archer explicitly specifies, based on a close reading of the transcripts and an understanding of how interviewees had navigated their way through the world. I did not use her Internal Conversation Indicator (ICONI) questionnaire to help me to categorize them.

## Reflexivity and charitable giving

This section will discuss how individuals with different dominant modes of reflexivity give to charitable organizations. However, fractured reflexivity is excluded from the analysis, because there were no individuals with predominantly fractured reflexivity in the sample.

### Communicative reflexives: Sociality and power

Twelve interviewees were identified as exercising largely communicative reflexivity, though its predominance varied among them. In several cases the communicative reflexives’ lived experience centres on caring and supporting children and elderly parents, and their personal identity is closely aligned to other people’s lives (Archer, 2007: 165–180). As a mother of two growing children, Mary, a former lower-middle-class legal secretary and now a part-time mature student, is busy with her children’s school runs and family visits:

I normally get up about 6, go for a run with the dog, come back, get the kids all sorted out, get their breakfast sorted out. I always like to give them a cooked breakfast, so I give them a cooked breakfast. While they’re eating that I go up and get showered, get changed, go down, take them to school, come [to the university], pick them up from school, start the dinner, take the dog for another walk, just do their homework. … I’ve got into the habit of coming to the university to sit in the library and do work because I found staying at home I ended up cleaning the house until 11.30 then I’d be hungry or might start get on the phone to somebody, so I was doing absolutely nothing to do with college work so if I’m out the house I actually crack on and get on with it. … Since the mid-term break, I’ve been just catching up with friends and I had both of my sisters over and they all stayed at Mum’s so I was down there every day, taking them out here, there and everywhere, so that’s three weeks of holiday.

Although Mary enjoys studying, it is not always a priority and has to fit into her hectic domestic schedule. Being a local part-time student allows her to be at university without having a negative impact on her family life. Some single parents have an intense relationship with their children. For instance, since her divorce, Jane, a working-class mature student, has developed a strong emotional attachment to her children:

We are very much together, and I love it, you go in and you go through the front gate and we are in our world. Me and the girls and sometimes children’s friends will come round but not that often and I love that, our little island. And it’s really quite nice, anti-social but it’s nice when we do want we want to do, because they are my little friends, really. So we are quite a little unit.

Jane is comfortable in her micro life-world, and values her relationship with her daughters. She is also cognizant that her daughters will leave home to study at university in the near future, and she will be alone. Attending a local university allows her to pursue her studies without severely affecting her family life.

There are two key interrelated elements of the communicative reflexives’ charitable giving. First, it tends to reinforce personal and social relationships, and not to be disruptive of a settled way of life (Eckstein, 2001). During the summer, James, a working-class estates supervisor, and his wife enjoy going to private gardens, especially those that support the Macmillan Cancer Support charity:

Macmillan Nurses we’ve always supported them because we go to the open gardens in the summer. … So it’s called ‘The Yellow Garden Scheme’, and the gardens are open, we’re lucky in Kent ‘cause there’s lots of them. So you go to a garden, it might cost you £2.50 to go in and you can also then buy teas and coffees and cake inside. What usually happens is the garden has to give a certain amount to Macmillan Nurses.

Visiting private gardens enables James and his wife to combine a family outing with charitable giving. As a result of buying tickets to the gardens, their visits contribute to charity. The Macmillan charity also has a special significance for James because it cared for his dying brother. James’s visits to charity gardens help to sustain family connections.

Some communicative reflexives view charities as an opportunity to socialize with others. For instance, Mary enjoys charity runs because they are an excuse for getting together with family and friends:

I always do a run for charity – part of that is fun as well because alright you are raising money for a good cause so that’s fantastic and everything, but y’know there would be a group of us running together, and we’d have a picnic in the park afterwards and, so it’s a social thing as well, so it’s not really a hardship. Sometimes it’s an excuse to do something, maybe we wouldn’t go for that run unless it was for a charity thing, … So it’s an excuse really to do something quite fun.

While Mary’s charity run will raise money for a good cause, it is also a social gathering that she shares with significant others. Without the social dimension, she and other communicative reflexives might possibly be less motivated to participate in charitable events. People’s moral motivations are rarely purely altruistic or self-interested, but rather mixed, part intrinsic, part instrumental (Sayer, 2010). Mixed motivations are quite common to all donors, and for some it is connected to improving their career prospects. Madeleine, a lower-middle-class estate agent, participates in a local Scout group to strengthen her curriculum vitae, and from a moral stance to help children from broken families:

It’s a fairly sort of some selfish motives for doing it. It would look good on my CV, which is one of the reasons for starting it, if I wanted to do that kind of career. Secondly, I genuinely think I can give children other experiences that they wouldn’t necessarily have and be a benefit to them. And I was just saying to my hairdresser before I left [to come here for the interview] that a lot of mothers always feel guilty that you’re not doing enough for your children or you look back at past events. I mean I was depressed for a number of years and probably wasn’t the best mother in the world, and I’m thinking maybe I can make up the shortfalls that I had with my children with other children that will somehow compensate.

In addition to having mixed sentiments of self-interest and compassion, Madeleine also has a strong emotional and sympathetic connection with other mothers, who like herself have to raise children in difficult personal and economic conditions. Smith (1976, VI.ii: 1–3) notes that sympathy and a feeling of moral responsibility are easier to elicit the closer others’ situations are to one’s own. Note also how Madeleine discusses her situation with her hairdresser, receiving confirmation of her reflexive deliberations.

Second, many communicative reflexives in the research tend to use social and moral norms to understand what causes are worth donating to and volunteering for. Moral discourses, stories and myths provide people with ways to think, shape and reason about how to treat and be treated by others, and the communicative reflexives usually accept such cultural constructs, rather than contest them. Several communicative reflexives give to well-known charities. For example, Paul, a former working-class commercial engineer now retired, notes:

For a very long time I’ve been making a regular monthly donation to a few chosen charities. The RSPCA [The Royal Society for the Prevention of Cruelty to Animals], I see them as the umbrella organization for all animal welfare, and I will not give money to any other animal charity on a regular basis. I do believe in the work of, like, Compassion in World Farming and these charities that look after, you know, old horses and greyhounds and things that – I cannot abide cruelty to animals. I also give money on a regular basis to the NSPCC [The National Society for the Prevention of Cruelty to Children], which again I see as an umbrella organization for children, I will also give to the Macmillan Nurses and Cancer Research and I consider that sufficient. . . . I’d also give, you know, to certain Christmas charities because I think, you know, there’s always a tradition in Britain that at Christmas you like to think of people who are not as fortunate as you. And when I was a child, when we had our Christmas dinner, my parents, you know, would always think of people who were less fortunate than ourselves, because I think it’s very important that you do that, you know, at least I think if you’re thinking about people it’s better than not thinking about them.

Paul has an emotional connection to causes about animals, children and cancer, reflecting the nation’s leading charity causes (see Mohan & Breeze, 2016: 28–32). He also donates to several major charities, which he views as umbrella organizations for their specific causes. In addition to his regular monthly contributions, Paul gives to several Christmas charities. For him, Christmas is an important and traditional time of the year to show generosity to needy and vulnerable groups, a message reinforced by his early family socialization of the ritual Christmas dinner as an occasion to reflect on less fortunate groups. Moral discourses and religious celebrations provide many communicative reflexives with rules to complete and confirm their internal deliberations.

In addition to inner dialogues, communicative reflexives rely on other people’s input to effect charitable giving. Family and social reactions to media appeals and street collections can spur individuals to donate, as Jane notes:

When they have the things on the telly like the tsunami, I mean [my daughters] were horrified, ‘Mummy, Mummy we have got to go and give them some money!’ … [My daughter] was only really quite small then and it was horrible that it affected them because you want to protect them from that kind of thing in the world. … When it’s in your face and I think it’s easier for everybody to give stuff that’s in your face.

Jane’s daughters pleaded with her to donate money after they watched a television appeal on the tsunami in Sri Lanka in December 2004. Public attention and pressure also help Jane to respond to ‘in your face’ street collectors and charity telethons. With large charities dominating the public space and the media, several communicative reflexives in the study support them.

The significance of other people’s participation in the communicative reflexives’ deliberations can leave the latter vulnerable to the former’s manipulation and pressure. Madeleine recounts how her local Scout group was threatened with closure, and the Scout committee persuaded her to become a manager to ensure its survival:

I sort of almost got cornered by the Scout leader and by the Chairperson of the committee who said, ‘Well there is no one to run it, so you could run it!’ ‘No, no I just wanna help out,’ I went. ‘What, run it? No, I wanna help.’ ‘Run it. If you run it, people will help.’ ‘But I think I’m the one saying I will help.’ ‘It’s only an hour a week, we’ll all help you. You’ll be alright.’ ‘Will I?’ ‘Yeah, yeah you will.’ ‘Ohh, I will. Well okay then, I’ll do it.’

Madeline was reluctant to manage the group, but gave in to the committee’s intense pressure, in part because she recognized that she and other mothers would benefit from its continuation. In general, morality can co-exist with power, since individuals’ self-interest and moral commitments can be exploited by others to further their own interests (Sayer, 2011: 177–179).

In the study, the communicative reflexives are more likely to accommodate themselves to prevailing social structures and norms, activating their powers to define their social relationships and charitable practices. For instance, while relations of care have many positive qualities, they are also strongly gendered, with women undertaking much of the burden of care (Tronto, 1994). Several female communicative reflexives’ volunteering involves gendered responsibilities of care and support. Madeleine reflects on how her maternal guilt moves her to become a ‘super-mum’, providing relief and help to other parents at the local Scout group:

I sort of think maybe there are other people going through those kinds of hard times and I would like somebody to be able to do that for my children, to be able to give them a good experience when I’m having a hard time. So if I can do it for other people’s children, then maybe because we have got children that come that obviously don’t always have the happiest of times so just for that one hour I can be super-mum or super-leader [at the Scout group].

In discussing their giving, Madeleine and Jane give attention to their own and others’ children, partly reflecting how their sentiments and roles are gendered and socially constructed. But their giving is also a normative and emotional response to the nature of their lives – their fragility, neediness and interdependency. Charitable giving is simultaneously an issue of power and morality.

### Autonomous reflexives: Instrumentality and moral individualism

Twenty-one interviewees were identified as exercising predominantly autonomous reflexivity to a varying degree. Their personal identity centres on career or practical worldly outcomes, obtaining personal satisfaction from their performance (Archer, 2007: 201–204). Most autonomous reflexives prefer to be mobile in order to have a good fit between their personal aspirations and their social context, opportunistically drawing on social and cultural resources to achieve their goals. Jackie, a lower-middle-class financial administrator, switched jobs twice in pursuit of better opportunities and advancements, and now enjoys the strategic decision-making part of her work:

My interest isn’t in real number-crunching, producing accounts and balance sheets and things like that, it’s very much more the forward looking, the planning, you know… If we, you know, particularly at the university, can assume we’re gonna get this many student numbers, that’ll bring in this much income, we’d need to run stuff at these costs, you know, that side of things is what interests me, the real sort of more strategic side of it.

Jackie also undertakes extra training and qualifications as she aims to develop her career. She wants to become a chartered accountant, and studies in the evenings for her professional exams. Many career-minded individuals are quite disciplined in dovetailing work with studies, family and friends, finding ways to accommodate different moral concerns. Peter, a working-class prison officer and a part-time mature student, deliberately chose The Open University degree, as it helps to fit together different things:

The Open University suits people like me I think, as well as various other people. It’s part-time. It suits me. I can listen to a lot of material and learn stuff in the car on the way to work. I can take my books into work while I’m supervising prisoners, or I might have a bit of down-time on my lunch break, so I can read through that. When I do get home and I’ve got some time, I can then type up my assignments. I don’t think it’s a case of seeing anybody less or spending less time with people, but I can have my friends over and I can still be studying while they’re over because I’m still spending quality time with my friends, although I might be doing other things, they’re still around me.

The Open University degree allows Peter to bring his studies into his work and home life without damaging his relationships with his employer and partner. Compare Peter’s approach to studies with Mary’s: Peter is more dedicated, seizing whatever opportunities he can to study.

There are two key interrelated elements of the autonomous reflexives’ charitable giving. First, it tends to have an instrumental value (see Curtis, 1997). Several autonomous reflexives use volunteering to strengthen their curriculum vitae. Peter believes that, when the prison service gets restructured in the near future, he may have to look for other employment. By volunteering as a special constable, he has a competitive edge over other applicants should he apply to join the police:

As a volunteer, you’re getting a background if you like into that before you make the full jump into being a police officer – you go through all the assessments and stuff. An average Joe off the street going for a job as a police constable, they don’t really know what it’s all about and they haven’t got the inside story if you like and they don’t know what’s going on, so it’s a bit of a risk, whereas, as a special, you get a bit of a background.

While most interviewees give morally mixed reasons for charitable giving, self-interest plays a more decisive role in motivating the autonomous reflexives than for the communicative and meta-reflexives. Compare Peter’s reasons for volunteering with Madeleine’s: he clearly elucidates how volunteering will serve his purpose of changing careers in the future. Some autonomous reflexives do not feel guilty about using charity in instrumental ways, but rather regard themselves as being alert and intelligent in knowing how to navigate through the system to get what they want.

Self-interestedness need not necessarily mean selfishness, as individuals can be compassionate on the basis of assessing their own vulnerability (Sayer, 2011: 119–124). Patrick, a working-class student and a part-time special constable, donates to an ambulance charity in the knowledge that one day he may have to use its life-saving services:

You do feel good about thinking the money you’re giving has potentially, you know, saved random people’s lives with regards to Kent Air Ambulance which one day I think, you never know, it might save my life. … But you just think, that you know, Kent Air Ambulance is there to support anyone and you never know, I might get run over one day on duty, it might come and pick me up and take me straight to a specialist hospital and might have saved my life. So I know by donating money it might save my life as well instead of other people’s.

Patrick’s donation to the ambulance charity reflects an element of enlightened self-interest. Should he get injured while on duty, the ambulance service may save his life. Several autonomous reflexives in the research contribute to civil society in the belief that they will gain from the transaction.

While many autonomous reflexives in the study value independence and privacy, they also require support and advice from others to make their way through the world. Some of them participate in charities when personal problems arise, drawing on their help and knowledge. When Mandy’s son was diagnosed with autism, her relationship with her parents became quite strained; she felt that they were unsympathetic and unsupportive. In addition, her marriage broke down under the strain of raising an autistic child. Feeling very lonely, Mandy, a working-class mature postgraduate student, managed to find some relief from her son’s challenging behaviour by volunteering at his school:

I found it therapeutic to be in [the children’s] company of how they should be acting, and actually enjoy the day and then I think I was kind of in the right frame of mind to deal with [my son’s] challenging behaviour, if that makes sense? I don’t know, but I think that’s why I found it enjoyable, is why I kept doing it, and things like, you know, going to assembly and even hearing them all sing and I did find it therapeutic… [The] voluntary work I think I was more helping myself. I knew I was helping the children because an extra pair of hands [in the classroom], and I felt good about having spent a day with them, but I think the main motivator probably was the therapy that I kind of felt from doing it.

Unable to properly communicate with her parents and her ex-husband, Mandy viewed volunteering as therapy to deal with her personal situation. In addition, she relied on a local support group of the National Autistic Society, which she donated to, for advice and assistance. But as Mandy became better at coping with her son’s autism, she stopped going to the local support group meetings and buying charity cards:

I did support the National Autistic Society with donation and Christmas cards, because it was something affecting me and I sort of did do it to try and keep them going because I thought they were really worthwhile. I did support them [when my son was diagnosed with autism], but then it kind of went a whole turnabout point where things sort of moved on, [and he] was in school. I think last year was the first time I didn’t buy the cards, because I kind of thought I don’t need to be sending everyone a card with NAS on it. I kind of felt that in my head I was okay with it.

When her son’s behaviour became manageable, Mandy ended her reciprocal relationship with the charity. Although she remains highly appreciative of its support, she does not feel obligated to carry on buying its charity cards, returning to her default position of moral individualism.

When charities do not have an instrumental value, some autonomous reflexives have spontaneous and ad hoc reflexive deliberations about them. Jackie is candid about her lack of reflexivity on the subject:

In all honesty it’s not a massive part of me … I don’t sit and feel, ‘Should I be doing something? I’m probably not pulling my weight’, or whatever it might be, if you see what I mean. ‘I could be doing more.’ … It’s just very ad hoc and it’s very much, like I say, reactive, as and when I’m asked for things I will do it. But it doesn’t matter enough to me, to be going out there, to try and find what else I could be doing.

Jackie does not get excited about giving, but will donate when asked to do so. Charity is not significant for her. She is much more reflexive about trying to dovetail her career and studies with her new family.

Second, many autonomous reflexives get personal satisfaction from their charitable performances and achievements. Terry, a former upper-middle-class naval officer now a semi-retired business owner, manages a local naval museum that allows former and retired naval officers, mechanics and engineers to restore de-commissioned sea vessels for public viewing at a local historical dockyard. When the naval dockyard closed down because of defence cuts, Terry and other former naval workers volunteered to put their skills into use to turn parts of the dockyard into a museum:

[When the dockyard closed] there were all these highly trained mechanics, communication specialists, engineers and so on. What do we do? Where do we go? … I saw the admiral, who was the chairman of the dockyard trust, and said, ‘We haven’t got a job anymore, do you want some of the lads and girls down here?’ He said, ‘Bring them down for volunteering!’ So we had about a hundred come in and then they all divvied up into different projects, we started on the submarine first of all, got it ready and open to the public, took two years. We had all sorts of different things for people to put their skills to – naval skills into other skills, museum-y type skills, you know, resurrecting things, restoring craft, ships, small-crafts, a helicopter. Someone went into archive work, someone into cataloguing photographs and books.

Terry has a feeling of self-satisfaction in the way the historical dockyard museum developed, from his initial proposal to the admiral to his managing ex-naval workers’ skills, resulting in retired war vessels being restored for public display. He feels proud of what he has accomplished, receiving national awards and honours in recognition of his work. Many autonomous reflexives engage with charities on their own terms, taking personal pleasure from doing charity and achieving success.

When personal satisfaction diminishes, most autonomous reflexives stop giving. Jimmy, a former lower-middle-class training consultant now retired, compares his charitable activities to hobbies that give him intrinsic pleasure, but he would stop doing them if they were no longer enjoyable:

It’s a self thing that you feel that you enjoy it, so why give it up if you enjoy it. One thing I would say about volunteering is I feel very comfortable about the fact, if I suddenly come to the point where I think to myself I’m not enjoying this any more, then I can just say sorry I’m not gonna do it any more, finished. I don’t have to give any reasons … I don’t necessarily have to feel guilty about it. All the time I feel that I personally enjoy doing it, if it helps somebody else along the way, then that’s a bonus, but I’m probably selfish in thinking the reason why I do it is because I like doing it, end of story.

Jimmy does not feel he needs to explain his reasons to others, nor does he feel guilty about being self-interested, content with his self-contained inner conversations on giving. Compare Jimmy’s self-determination and lack of guilt about volunteering to Madeleine’s submission and guilt. Moral individualism is a salient feature of many autonomous reflexives’ accounts of charitable giving, expressed in terms of self-responsibility (Peter), self-interest (Patrick), self-help (Mandy), self-satisfaction (Terry) and self-determination (Jimmy). It partly articulates their lay understanding of morality, drawing on available cultural resources and scripts, and is shaped by historical and structural conditions, such as the ideology of competitive individualism, the neoliberal discourse of the enterprising self, working-class aspirations for social betterment and upward social mobility, and middle-class dispositions towards career advancement and capital accumulation.

### Meta-reflexives: Social criticisms and politics

Eight interviewees were identified as predominantly exercising meta-reflexivity to a varying degree. The meta-reflexives have a deep commitment to moral, religious and political beliefs, and have a strong sense of compassion and social justice (Archer, 2007: 261–265). Some of them challenge specific social arrangements that are incongruent with their values, aspiring to lead a complete ethical life. Sophie, a lower-middle-class researcher, is passionate about protecting the eco-system, abhorring its abuse:

I just feel that my purpose on this planet is to help and protect animals and every day when I leave for work to catch a bus, if it’s been raining all the worms are up on the ground and I can’t walk by and step over them or whatever. I move every single worm, every single slug and snail that I see along the road as I get to the bus stop. . . . And as far as I’m concerned if it’s living, then it has a right to live and we don’t have the right to kill it. I will rescue anything I can to give it the opportunity to live. [In my local supermarket], I’ve got a whole campaign, in fact they dread me coming in now, because they are failing to water their plants regularly. So when I go in, I insist on the manager coming, pointing out which ones want watering … I just feel that God has put me on this planet to sort of protect animals and to some degree plants and stuff like that.

Sophie has intense reflexive deliberations about animal welfare and plant life. Although she is withdrawn and reticent in work and social situations, she is quite forthright and confident in helping animals and plants, going to extraordinary lengths to intervene on behalf of non-human living things, in the belief that it is her ‘calling’. In the past she has rescued various animals from neglect and harm, and has started a local campaign to stop developers from destroying primroses and wild orchids, and chopping down trees. She also refuses to donate to specific charities that experiment on animals.

Some meta-reflexives are deeply reflexive about major social and political issues (e.g. homelessness and the environment) and personal matters. William, an upper-middle-class lecturer, agonizes over the ethics of his undeclared income:

I have some additional income from various little bits and bobs, things like teaching piano lessons and so on, and you know, part of me thinks, it would be very easy, I get paid in cash, you know just have this little bit of extra income, not to mention it to the tax people and so on, and obviously then it’s a moral question of, ‘Do you alert the tax people to what is a comparatively modest part of your income and thereby see 20 per cent of it disappear again?’ … I debate the issues and say, you know, ‘What do I want to do here? What is the right thing to do? Does it matter?’

William embarks on a lengthy internal conversation, mulling over his ethical dilemma. He also describes another inner dialogue about the cost of shopping for ethical and organic products.

While religious faith is an important reference for several meta-reflexives, it usually amalgamates with political and moral discourses to shape their values and beliefs. For instance, Sophie’s worldview consists of conservative Christianity and veganism, for William it’s liberal Christianity and environmentalism, for Kamela liberal Christianity and egalitarianism, and for Geraldine liberal Christianity and anarchism. Some meta-reflexives are atheists and draw on secular discourses, such as humanism and liberalism.

There are two key interrelated elements in the meta-reflexives’ charitable giving. First, charitable giving is likely to be viewed as a matter of compassion and social justice. Eve, a working-class part-time hospital porter, gives to Shelter, a homeless charity, because homeless people are vulnerable and blameless for their situation:

For example, a single mother with three children whose other half batters her. She will have to go and get temporary housing to be housed away from him and then she’ll be left in there for two year. That’s not her fault. … That’s just a bad situation, bad circumstances, bad luck really and a lot of the people who are homeless, it’s just bad luck that’s befallen them and so they need help really and the government doesn’t help them and they fall through the net, so and it’s sad and not enough people really care about it as far as I can see. There’s too much of this, kind of, it’s their own fault sort of idea.

She has sympathy for homeless people, having been homeless herself. It is common for most donors to sympathize with others who have suffered misfortunate or injustice, having experienced or imagined similar or related forms of suffering. But the meta-reflexives tend to point out that social institutions are partly responsible for social problems. Eve is angry that the government does not provide sufficient temporary and emergency accommodation. She is also critical of the way the media often blame homeless people for their own plight without understanding how bad luck affects their lives, some coming from broken and abusive homes for example.

Of course, recognition of injustice and suffering can come from political and moral beliefs as well as from personal experience. Several meta-reflexives have intense reflections on what values should guide them and society (Archer, 2003: 288–295). Kamela, an upper-middle-class information technology manager, volunteers for the Independent Monitoring Board, which protects prisoners’ rights. She is critical of how the prison system unfairly treats prisoners:

I suppose prisoners are not popular are they, you know, it’s very difficult to get sympathy for prisoners, yeah I find it so frustrating when people moan about that fact that they have TVs in their cells, like a four star hotel. … [Prisoners] obviously have rights taken away from them, that’s why they’re in prison, but in terms of being treated with respect and humanely they have, you know, the same rights as any of us, and if me going in there ensures that happens, that they get treated humanely, and with respect, then that’s worth it. If they are being treated humanely and with respect while they are inside prison gives them the opportunity to turn their lives around when they come out because they are given some sense of worth because of their treatment, then that has to be a real plus.

Kamela has strong views on equal rights and human dignity, and believes that prisoners should be treated humanely and with respect. She ensures that prison rules are correctly followed, and that prisoners are given ample opportunities and encouragement to lead a better life once they are released. She is scornful of attempts by right-wing politicians and the media to hinder humane penal reforms.

Some meta-reflexives have a strong sense of moral obligation verging on supererogation that involves significant personal sacrifices. Eve’s donations to Shelter mean that she struggles to pay her local council tax and utility bills, but nevertheless she feels comparatively well-off:

I am passionate about Shelter yeah. I know that there are people out there that are worse off than me … I guess people who are in sort of privileged positions, i.e. have a house, have good education, have enough to get by on, have a job, have money and generally happy, it’s really kind of our responsibility that other people do have, who aren’t as fortunate, that they can have some kind of happiness.

Feeling relatively privileged, Eve makes monthly donations to the homeless charity and tithes to her local church, which uses some of its income to manage an accommodation centre for the homeless. When walking down the street at night, she often stops to talk and give cigarettes to people sleeping out, some of whom she knows from her own turbulent past. Although her partner gets upset that she makes excessive contributions when they do not have enough food on the table or enough money to heat the house, she feels duty-bound to tithe and make donations, and usually gives cigarettes to the homeless without her partner’s knowledge.

Second, in criticizing existing social institutions, most meta-reflexives have a different vision of society, and generosity plays a significant role in creating a fairer society, fostering values of human interdependence, obligations and well-being (see Smith & Davidson, 2014: 53–86). Kamela believes that, although generosity can have some negative results, it is better than being cynical:

Sometimes you have to give first, and sometimes you have to give and get kicked in the face for doing it and still give … I’d still rather carry on being that than being cynical and bitter, because that not only affects everybody else around me and their quality of life if I’m cynical and bitter, but it sure as hell affects mine, you know, my outlook on life … Because no man’s an island. You know we’re all interconnected, and most of us wanna go through life with people being nice to us, and not being sort of cynical and thinking ‘Oh, I’m alright Jack!’

For Kamela, individuals are interdependent and needy beings, who have to rely on others for love, care and support. She suggests that an excessive focus on the self and the family can threaten social solidarity and human well-being, and laments how people’s lives are often sacrificed for profits:

We’re not gonna have the best society we can, unless people are prepared to give … We went a lot wrong in our society in the Thatcher years, when everybody thought it was okay to make lots of money and ‘I’m alright Jack, and stuff you!’, and what does it matter if I make a fortune on the back of three million unemployed, you know, I’m okay, my family’s okay. You know, it really annoys me when people say ‘Charity begins at home.’ What you mean just within that four walls, you know, what is home? Home’s big? You know, I believe in communities, I believe in church communities, school communities, you know, guides and cubs and that sort of thing … So I just think we’re a poorer society if we don’t give.

Kamela’s charitable giving is framed in terms of communal and collective values. Whereas many autonomous reflexives (such as Patrick and Mandy) view giving in personal and strategic terms, Kamela highlights its social transformative effects. She feels that there are not enough people donating and volunteering, because they are too individualistic and materialistic. She regularly donates money to her parish church, and helps with fundraising activities at her local school and church fêtes.

Some meta-reflexives undertake active roles in civil society, hoping to change social relationships and institutions to ensure a better treatment of disadvantaged communities. But when their goals have been satisfied or their ideals are no longer compatible with their charities, they leave for another charity. Geraldine, a lower-middle-class mature student, was influential in starting up a self-advocacy group for people with learning disabilities in her local community. The group was successful, expanding its membership and winning local council grants to run training programmes. But after two years, Geraldine had achieved her goal of empowering the group members and had nothing more to offer:

[We were] supporting people with illiteracy, supporting people with having the confidence to speak in front of people, supporting people to learn things like how do you run a meeting, and what sort of behaviours are appropriate when you go in a big meeting and, for example, going up to London, supporting people on the Tube because that’s something that they couldn’t necessarily do. And also at the same time, doing yourself out of a job. The main thing you have to do is know what the ideal is. At the end of two years, they don’t really need you. And I don’t know whether the project director who runs the group would agree or not, but I thought we got there at the end of last year. That they really didn’t need a supporter/carer any more.

At the start Geraldine was delighted with her role as a supporter and carer within the group, empowering and meeting the needs of the group. After a couple of years, she felt that her goals were accomplished, and left the organization. Compare her reason for leaving the charity with Mandy’s – Geraldine doubted that she could make any further meaningful contribution to the charity. She now volunteers for Médecins Sans Frontières, which she praises because it provides medical assistance to people living in dangerous places and is critical of foreign governments.

## Addressing some criticisms

Some readers may be dissatisfied with the three-stage model of reflexive donors and volunteers, criticizing the model for: (1) neglecting to say how social and cultural structures can shape, or correlate with, the different modes of reflexivity; (2) failing to contextualize reflexive deliberations in different social settings; (3) exaggerating the role of reflexivity in daily life and underestimating the influence of socialization, dispositions and habits; and (4) ignoring the messiness and complexity of social life by neatly fitting interviewees into Archer’s ideal-types. I will briefly address each of these criticisms.

First, in this article, the different modes of reflexivity are analysed as causes of specific courses of action, and the article does not examine the effects of social and cultural structures on reflexivity. The three-stage model of reflexive donors maintains that the modes cannot be reduced to social factors, but rather emerge from social and psychological processes that have their own distinct properties and powers. In proposing the model, I highlight how reflexivity is an important and neglected dimension of social and charitable practices, while noting that social factors (like gender, class and discourses) are also relevant.

It is tempting to correlate the working class with communicative reflexives, the lower middle class with autonomous reflexives, and the upper middle class with meta-reflexives. It also seems plausible that class can shape reflexivity, with the working class more likely to accommodate and resign themselves to existing social structures and, lacking cultural and social capital, likely to occupy passive roles in civil society. In contrast, the middle class are more likely to have the economic and cultural resources to articulate social criticisms and to fill commanding roles in charitable organizations to envision social change. It is also tempting to suggest that gender can shape reflexivity, with women more likely to be communicative or meta-reflexives because they undertake care roles and responsibilities in the family and community, and are sympathetic and attentive to suffering. In contrast, men are likely to be autonomous reflexives because they are more individualistic and focused on achievements, and have more distant and instrumental relationships. But reflexivity cannot be reduced to social structures because it emerges from social and psychological processes, and has distinct properties and powers from structure and agency. While knowing people’s social position can be a good indicator of likely behaviour, it is never enough to give a complete understanding of how they interpret their situation in relation to their concerns – this requires an account of their reflexivity. Without reflexivity, people lack the capacity to deliberate, evaluate and change their circumstances. Ironically, in Bourdieu et al.’s (1999) *The Weight of the World*, the interviewees discuss and evaluate the legitimacy of their situations and the dominant discourses about them. Sayer (2005: 27–30) argues that accounts of social practices need to recognize both the scope of unreflective practical action and the role of conscious monitoring and deliberation in everyday life.

Second, although the article focuses on the interviewees’ dominant mode of reflexivity, it does acknowledge that they can also engage in other modes. They can have intense and critical reflexive deliberations in certain social situations, fleeting and spontaneous ones in some, and act unconsciously in others. The article analyses how they have different modes of reflexivity about charitable giving, rather than exploring their reflexive deliberations and habits across different social contexts.

Third, the model recognizes that it is not always necessary for people to discursively articulate and justify their actions or to possess a penetrating discursive understanding of their situations; all they need to recognize consciously and find good is the ease and fluency of their actions. Furthermore we may be suspicious of individuals who always intensely deliberate before acting; love, care and professionalism are valued partly because they are spontaneous and stable. In addition, the article does discuss the different ways in which socialization, power and ideology can shape charitable giving, noting the effects of gendered roles and moral individualism on giving.

Fourth, it is incorrect to say that the data merely fit into Archer’s analysis. Contrary to her expectations, the research found that all but one of the reflexive groups are involved in charities. This partly reflects the point noted above, that the differences among the three groups often lie in degrees rather than in kind, and that there are some important similarities among them. Furthermore, in explaining charitable giving, the article recognizes the need for both conscious monitoring and socialization.

## Conclusion

I have examined how charitable giving has different meanings and significance for the three groups of reflexive donors and volunteers. For many interviewees who largely exercise communicative reflexivity, charities give them an opportunity to do good work with and for others, some of whom are significant figures in their lives. Giving is seen as a communal activity that establishes and affirms social connections and norms. For most participants with predominantly autonomous reflexivity, charities are useful for achieving practical things that matter for their well-being. Moral individualism is a salient feature of their deliberations about giving. For several interviewees exercising largely meta-reflexivity, charitable giving is about making society more humane and fair by addressing social problems and issues. Giving is viewed as helping to create a different world based on human dignity and respect.

But the study also found that the three groups share some important elements that shape their charitable giving. These include the capacity to sympathize and understand others’ suffering and misfortunes, mixed sentiments of self-interest, compassion and justice, and moral discourses and rules. The differences among the three groups often lie in degrees rather than in kind. For instance, while instrumentality and reciprocity strongly influence most of the autonomous reflexives’ decisions about charities, they also affect other groups’ deliberations. Sociality and conventions are significant in explaining many of the communicative reflexives’ giving practices, but are also present in the deliberations of most autonomous reflexives and meta-reflexives. For almost all the meta-reflexives, giving reflects their desire and longing for a different world, sentiments that are also shared with many communicative and autonomous reflexives. It is worth repeating that most people engage in one or more modes of reflexivity over the course of their lives, though one of them tends to be dominant at any particular stage. Although the dominant mode is likely to be stable, it is not fixed, because it changes in response to social and personal dynamics, including illness, children leaving home, divorce, education and unemployment.

Finally, the study rejected the standard two-stage model of structure and agency, arguing that much of contemporary sociology tends to conflate the distinct structural and agential properties and powers. This often produces reductionist (and cynical) accounts of social practices that usually deny people’s capacity to evaluate and change either themselves or their circumstances. Instead, the article offers the three-stage model, in which internal conversations mediate the impact social structures have on agents’ concerns, and condition individual responses to particular social situations. This offers a better understanding of how individuals interpret their situation in relation to their concerns. In explaining everyday practices, however, accounts need to recognize both deliberations and unreflexive practical action.
